# Long-Term Trends Worldwide in Ambient NO_2_ Concentrations Inferred from Satellite Observations

**DOI:** 10.1289/ehp.1409567

**Published:** 2015-08-04

**Authors:** Jeffrey A. Geddes, Randall V. Martin, Brian L. Boys, Aaron van Donkelaar

**Affiliations:** 1 Department of Physics and Atmospheric Science, Dalhousie University, Halifax, Nova Scotia, Canada; 2 Harvard-Smithsonian Center for Astrophysics, Cambridge, Massachusetts, USA

## Abstract

**Background:**

Air pollution is associated with morbidity and premature mortality. Satellite remote sensing provides globally consistent decadal-scale observations of ambient nitrogen dioxide (NO_2_) pollution.

**Objective:**

We determined global population-weighted annual mean NO_2_ concentrations from 1996 through 2012.

**Methods:**

We used observations of NO_2_ tropospheric column densities from three satellite instruments in combination with chemical transport modeling to produce a global 17-year record of ground-level NO_2_ at 0.1° × 0.1° resolution. We calculated linear trends in population-weighted annual mean NO_2_ (PWM_NO2_) concentrations in different regions around the world.

**Results:**

We found that PWM_NO2_ in high-income North America (Canada and the United States) decreased more steeply than in any other region, having declined at a rate of –4.7%/year [95% confidence interval (CI): –5.3, –4.1]. PWM_NO2_ decreased in western Europe at a rate of –2.5%/year (95% CI: –3.0, –2.1). The highest PWM_NO2_ occurred in high-income Asia Pacific (predominantly Japan and South Korea) in 1996, with a subsequent decrease of –2.1%/year (95% CI: –2.7, –1.5). In contrast, PWM_NO2_ almost tripled in East Asia (China, North Korea, and Taiwan) at a rate of 6.7%/year (95% CI: 6.0, 7.3). The satellite-derived estimates of trends in ground-level NO_2_ were consistent with regional trends inferred from data obtained from ground-station monitoring networks in North America (within 0.7%/year) and Europe (within 0.3%/year). Our rankings of regional average NO_2_ and long-term trends differed from the satellite-derived estimates of fine particulate matter reported elsewhere, demonstrating the utility of both indicators to describe changing pollutant mixtures.

**Conclusions:**

Long-term trends in satellite-derived ambient NO_2_ provide new information about changing global exposure to ambient air pollution. Our estimates are publicly available at http://fizz.phys.dal.ca/~atmos/martin/?page_id=232.

**Citation:**

Geddes JA, Martin RV, Boys BL, van Donkelaar A. 2016. Long-term trends worldwide in ambient NO_2_ concentrations inferred from satellite observations. Environ Health Perspect 124:281–289; http://dx.doi.org/10.1289/ehp.1409567

## Introduction

Globally, > 3 million premature deaths were attributed to ambient air quality in 2010 (Lim et al. 2012), and air pollution has been estimated to cost the United States $71–277 billion in gross annual damages primarily as a result of premature mortality and illness (Muller and Mendelsohn 2007). Decades of large cohort studies have established strong associations between air pollution and human mortality, with increasing evidence of a “no-threshold” model where adverse health effects can be recognized even at low ambient levels [Anderson 2009; World Health Organization (WHO) 2006]. As air quality around the world evolves, global observations of these long-term changes would offer valuable insight into trends in exposure.

Nitrogen dioxide (NO_2_) is a major constituent of the air pollution mix. Strong associations between NO_2_ and mortality have been identified in multicity studies around the world (Burnett et al. 2004; MacIntyre et al. 2014; Samoli et al. 2006; Tao et al. 2012). There is inconsistent evidence for a mechanistic driver of the effects of NO_2_ on health (Hesterberg et al. 2009); thus, it is unclear whether NO_2_ acts as an independent cause of mortality. Rather, there is consensus that NO_2_ could act as a proxy or surrogate species either for constituents that are not being monitored, or more generally for the multipollutant mix (Brook et al. 2007; Brunekreef and Holgate 2002; Levy et al. 2014). Thus, NO_2_ has been included in multipollutant health indices (Stieb et al. 2008) and is strictly monitored in some regions (WHO 2006).

Given the robustness of NO_2_ as a predictor of the effects of air pollution on health, there is a great deal of interest in long-term estimates of historical concentrations. However, few regions, especially in developing countries, have collected sufficient observations for exposure assessment. In a few parts of the world, ground-station monitoring networks have operated for decades, providing a valuable record to evaluate satellite-based trends in air pollution.

Seltenrich (2014) summarized the opportunities offered by remote sensing for environmental health research in a recent Focus article in *Environmental Health Perspectives*. Novotny et al. (2011) and Vienneau et al. (2013) combined satellite data and land-use regression to calculate population-weighted mean NO_2_ over the United States in 2006 and over western Europe in 2005–2007. Novel applications have been found for such data in exploring disparities in exposure by socioeconomic factors (Clark et al. 2014). Observations of tropospheric NO_2_ vertical column densities from satellite instruments have provided evidence of dramatic changes over the United States (Duncan et al. 2013; Russell et al. 2012) and around the world (Hilboll et al. 2013; Richter et al. 2005; van der A et al. 2008). These results show the potential to document globally consistent changes in total atmospheric burden, but the observations have yet to be extended to long-term surface-level concentrations.

Here, we inferred annual mean ambient ground-level NO_2_ concentrations worldwide from 1996 to 2012 using observations of NO_2_ tropospheric column density from three satellite instruments together with a chemical transport model. We investigated trends in regional population-weighted mean NO_2_ concentrations over these 17 years and contrasted our results with similar published estimates for fine particulate matter (PM_2.5_).

## Methods

*Satellite observations.* We used NO_2_ column observations from the Global Ozone Monitoring Experiment (GOME), Scanning Imaging Absorption Spectrometer for Atmospheric Chartography (SCIAMACHY), and GOME-2 satellite instruments. Information on these satellite instruments is available at Earth Online (https://earth.esa.int/). We chose these instruments for their similar observation times (typically 0930–1130, 0900–1100, and 0830–1030 hours local time, respectively), with some temporal overlap owing to the range of viewing angles. Data were obtained from the Tropospheric Emission Monitoring Internet Service [http://www.temis.nl/airpollution/ (TM4NO2A, v2.3)]. The NO_2_ column densities were retrieved from measurements of backscattered sunlight (Boersma et al. 2004). The GOME instrument provided observations from 1995 to 2003, with a spatial resolution of 320 km × 40 km, and covered the globe roughly every 3 days. SCIAMACHY provided observations from 2002 to 2011, with a spatial resolution of 60 km × 30 km resolution, and covered the globe in approximately 6 days. GOME-2 has observed since 2007, and continues to do so, with a spatial resolution of 80 km × 40 km, covering the globe in one day. We regridded the daily data from each instrument to a regular 0.1° × 0.1° grid worldwide. This approximately 10 km × 10 km resolution is finer than any of the individual instruments but allows for direct comparison between them. Detection of smoothed features at scales below instrument pixel dimensions can occur for long-term mean quantities because of spatial oversampling (Streets et al. 2013).

We excluded data contaminated by cloud or snow by rejecting bright pixels (cloud radiance fraction > 0.5) and by filtering data according to the shortest snow-free season [estimated by marking the last day of snow cover and the first day of snow arrival in each pixel for every year, using data from the National Ice Center’s Interactive Snow and Ice Mapping System (http://dx.doi.org/10.7265/N52R3PMC)].

We accounted for the retrieval sensitivity by replacing the *a priori* NO_2_ profile assumed in the retrieval using the provided averaging kernels with the data, together with daily mean profiles for between 1000 and 1200 hours obtained from a chemical transport model (GEOS-Chem) simulation, following Equation 1 of Lamsal et al. (2010).

*Estimating ground-level NO_2_.* We used the GEOS-Chem model (Bey et al. 2001), v9-01-03 (http://geos-chem.org), to simulate the relationship between satellite observations of tropospheric NO_2_ column densities and the NO_2_ concentrations at ground level relevant to human exposure following the method described by Lamsal et al. (2008, 2010). GEOS-Chem is a freely accessible community global chemical transport model that solves for time-varying three-dimensional atmospheric composition using equations that represent the chemistry and physics of the atmosphere. We conducted simulations for January 1996 to December 2012 at a horizontal resolution of 2° latitude by 2.5° longitude with 47 vertical levels (14 within the lowest 2 km); we used assimilated meteorological fields [Modern-Era Retrospective Analysis for Research and Applications (MERRA)] provided by the National Aeronautics and Space Administration’s (NASA’s) Global Modeling and Assimilation Office. Details of the simulations (e.g., emission inventories) have been described by Boys et al. (2014).

*Reconstructing consistent spatial resolution.* The different horizontal resolution from the three satellite instruments has hindered the use of NO_2_ satellite observations for long-term pollution studies. One approach to deriving a self-consistent record over the three instruments is to degrade higher-resolution observations to a single consistent coarse resolution [e.g., van der A et al. (2008) and Konovalov et al. (2010)], but this method forfeits the spatial information available from SCIAMACHY.

Here, we simulated fine spatial structure in both the GOME and GOME-2 observations by assuming that the relative spatial structure observed during the SCIAMACHY time period persists during the GOME and GOME-2 time periods. This approach exploits the general persistence over multiple years of locations where NO_2_ is produced. Similar approaches have been used for long-term trends in satellite observations of tropospheric NO_2_ (Hilboll et al. 2013; Konovalov et al. 2008). We smoothed SCIAMACHY data using a two-dimensional boxcar algorithm with an averaging window of 3.2° × 0.4°, roughly reflecting the horizontal smearing of the GOME resolution of 320 km × 40 km. We treated the smoothed SCIAMACHY data as a reproduction of GOME observations (global land-covered pixel-by-pixel scatter between mean SCIAMACHY and GOME was correlated with *r* = 0.92 during their period of overlap from 1 August 2002 to 30 June 2003). We subsequently downscaled the annual mean GOME observations from 1996 to 2002 by applying the ratio of temporally averaged high-resolution (0.1° × 0.1°) observations in SCIAMACHY from 2003 to 2005 to the averaged smoothed (3.2° × 0.4°) observations. Figure S1a,b in the Supplemental Material shows the results over North America for the year 1999. Urban centers smeared by GOME are resolved by SCIAMACHY.

This approach assumes that *a*) the relative spatial gradients of NO_2_ do not change significantly over the GOME time period, and *b*) the spatial gradients in NO_2_ that were observed in 2003–2005 were representative of 1996–2002. We evaluated these assumptions during a period of rapid change in NO_2_ columns (see Supplemental Material, Figure S1c,d): we smoothed the observations from 2009 to 2011 using the same two-dimensional boxcar algorithm (see Supplemental Material, Figure S1e), then downscaled the results using the high-resolution observations from 2003 to 2005 (see Supplemental Material, Figure S1f). The true observations from 2009 to 2011 (see Supplemental Material, Figure S1d) were significantly correlated (*r* = 0.97 over all land-covered pixels globally) with the simulated observations (see Supplemental Material, Figure S1f). The correlations were also significant over specific domains where NO_2_ columns have changed rapidly, including North America (*r* = 0.96), Europe (*r* = 0.96), and Asia (*r* = 0.98).

We interpret these correlations as confirmation that our assumption of the general persistence in combustion source areas can plausibly be extended back during the GOME record. Although the observation resolutions of SCIAMACHY and GOME-2 were more similar, we found that a correction was still required to produce a self-consistent record. In this case, the reconstruction was based on a more direct comparison. The long-term ratio (2007–2011) of SCIAMACHY to GOME-2 data was applied to each GOME-2 annual mean with the assumption that scaling would be constant until 2012. The result is a self-consistent data set at the effective resolution of SCIAMACHY for 1996–2012. After the spatial correction, we found significant agreement during overlapping periods between GOME and SCIAMACHY (*r* = 0.89) and GOME-2 and SCIAMACHY (*r* = 0.95). To further test for systematic evidence of a discontinuity or bias between sensor records, we calculated *z*-scores for the difference in NO_2_ between all neighboring years in the record of the individual pixels for major cities worldwide. The difference in NO_2_ between 2003 (GOME) and 2004 (SCIAMACHY), and between 2011 (SCIAMACHY) and 2012 (GOME-2), were within 1.5 standard deviations for 94% of cases.

*Evaluation using ground-based observations.* We evaluated the satellite-derived product by comparing time series over 10 densely populated regions in North America and Europe with multiple continuous ground monitors from 1996 to 2012 within the surrounding ~ 200 km × 200 km area. We focused on relative changes given the large spatial variation in absolute NO_2_ concentrations within these areas (Jerrett et al. 2007). We also evaluated our satellite-derived estimates of gridded NO_2_ concentrations by comparing the long-term mean observations from the collocated ground-station monitors. Hourly observations of NO_2_ across North America and Europe were obtained from the U.S. Environmental Protection Agency (http://www.epa.gov/aqs), Environment Canada (http://maps-cartes.ec.gc.ca/rnspa-naps/data.aspx), and the European Environment Agency (http://www.eea.europa.eu/data-and-maps). The NO_2_ recorded by these monitors is known to suffer from interference by other reactive nitrogen compounds, resulting in a location-specific overestimate. Differences in representativeness between ground-station and satellite data also arise from comparing a point measurement with an area average, magnified by the tendency for ground monitors to be in locations with elevated NO_2_ concentrations. We therefore focused on evaluating relative trends.

We calculated midmorning (1000–1200 hours) average observations each day. We excluded European stations identified as “traffic” to maximize site representativeness at the 0.1° × 0.1° scale (no such identifier was available for the North American networks) but otherwise retained all rural, suburban, and urban locations (represented roughly equally). We required stations to have at least five satellite-coincident observations per year and at least 15 years of observations. Observations from multiple stations within a single pixel were averaged. We used completely sampled ground-based annual averages to evaluate the satellite-derived record.

*Annual means and long-term trends.* Incomplete sampling from the satellite instruments may introduce biases in long-term averages where NO_2_ concentrations are correlated with season or cloudiness. We accounted for this potential bias by using a pixel-dependent correction, calculated as the annual ratio of GEOS-Chem simulated surface NO_2_ sampled each day versus only on days with successful satellite retrievals. This approach has been used previously for analyses of NO_2_ (Nowlan et al. 2014) and PM_2.5_ (van Donkelaar et al. 2010).

We calculated long-term trends in annual means by ordinary least-squares linear regression with 95% confidence intervals (CIs) to establish uncertainty in the slopes. Results were considered statistically significant at α ≤ 0.01. We report trends in percent/year relative to the long-term mean. We compared concentrations and trends in NO_2_ with previously published values for PM_2.5_.

*Population data.* We obtained worldwide gridded population counts (available at 5-year intervals from 1995 to 2015, with 2.5-arc-minute resolution) from the NASA Socioeconomic Data and Applications Center [Gridded Population of the World (GPW) v3; http://sedac.ciesin.columbia.edu/]. We aggregated the data to 0.1° × 0.1° and linearly interpolated between each interval. We used these data to calculate time series in population-weighted annual mean NO_2_ (PWM_NO2_) for the same regions as in the Global Burden of Disease (GBD) Study 2010 (Lim et al. 2012; http://www.healthdata.org/gbd/).

## Results

**Figure 1:**
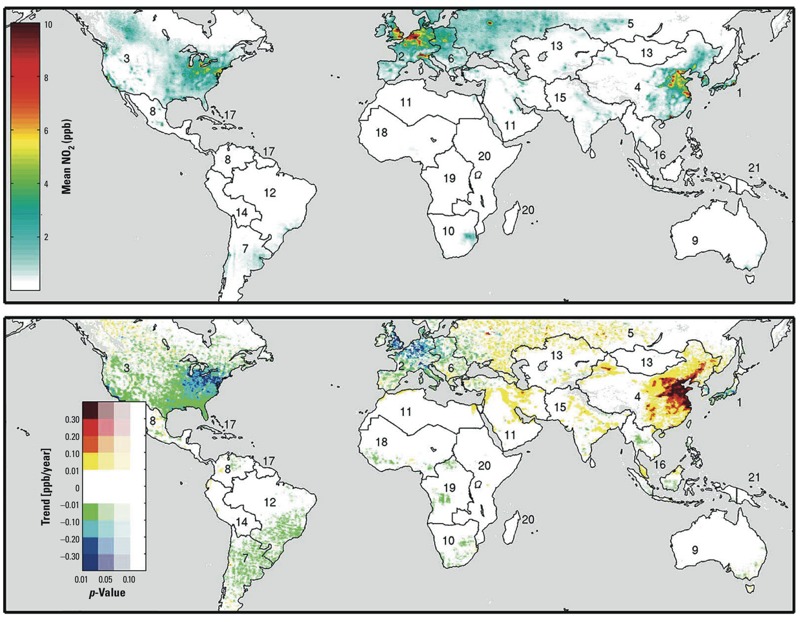
Long-term (1996–2012) mean satellite-derived ground-level NO_2_ concentrations (top), and trends in NO_2_ over the same period (bottom). Numbered regions and their boundaries correspond to the region names and numbers given in Table 1.

Figure 1 shows the satellite-derived long-term means and trends in ground-level NO_2_ worldwide. We have made these data publicly available at http://fizz.phys.dal.ca/~atmos/martin/?page_id=232 (“Surface NO_2_”). The most notable reductions were observed in North America, western Europe, and Japan. In contrast, ground-level NO_2_ substantially increased in East Asia and in isolated urban regions of the Middle East, Russia, and India. Trends for specific urban areas around the world are clearly distinguishable. For example, the steep increase isolated in northwestern China is around the city of Ürümqi (43.8°N, 87.6°E), where NO_2_ increased at a rate of 10.1%/year (95% CI: 8.0, 12.1).

The satellite-derived product was significantly correlated in space with the coincidently sampled ground-station observations in North America (*r* = 0.80, *n* = 223). The correlation was slightly weaker across rural locations (*r* = 0.72, *n* = 41) than across nonrural locations (*r* = 0.81, *n* = 181). We found that the satellite-derived product for Europe was less strongly correlated with ground-station observations (*r* = 0.57, *n* = 365) than that for North America, but in this case, rural locations were highly correlated (*r* = 0.81, *n* = 120), and non-rural locations were less so (*r* = 0.60, *n* = 245). These correlations are consistent with previous comparisons of satellite-derived NO_2_ with NO_2_ data from monitoring networks (Blond et al. 2007; Lamsal et al. 2008, 2010). We attribute the variation between rural and nonrural stations to differences in the spatial representativeness of the sites, with urban monitors influenced by local pollution (e.g., traffic).

**Figure 2:**
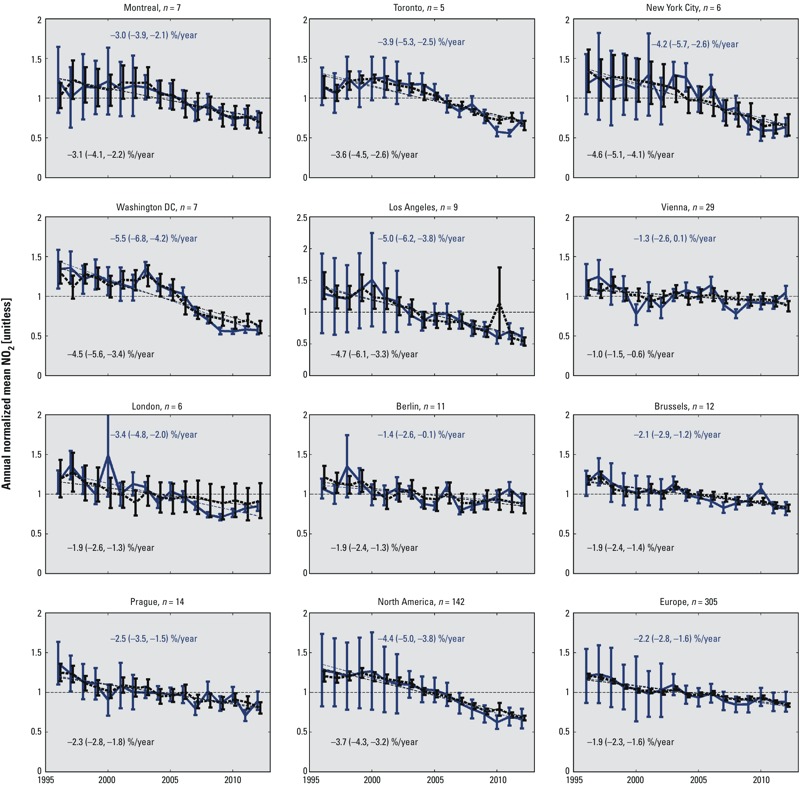
Annual mean satellite-derived (solid blue lines) and ground-based (dashed lines) NO_2_ concentrations over North American and European urban areas, normalized to the respective long-term means. *N* denotes the number of ground-based stations within the urban area. Linear trends are reported as percent/year with 95% confidence intervals. Error bars denote the standard error of the annual ground-based measurements or the standard error of satellite pixels within the area.

Figure 2 shows time series of spatially averaged annual mean ground-level NO_2_ normalized to the long-term mean over 10 urban areas with continuous observations. The majority of the trends in satellite-derived and ground-based mean NO_2_ are in accord within the 95% CIs. The last two panels of Figure 2 show satellite-derived NO_2_ time series averaged over all North American (*n* = 142) and European (*n* = 305) pixels with continuous ground-station measurements compared with mean ground-station measurements. The linear trends for the satellite estimates across North America of –4.4%/year (95% CI: –5.0, –3.8) and across Europe of –2.2%/year (95% CI: –2.8, –1.6) are consistent with the ground-based measurement trends of –3.7%/year (95% CI: –4.3, –3.2) and –1.9%/year (95% CI: –2.3, –1.6), respectively.

*Population-weighted annual mean NO_2_.* To our knowledge, this is the first data set that allows estimates to be made of long-term global changes in area-wide ambient NO_2_ concentrations relevant to outdoor exposure. Table 1 summarizes the long-term means and long-term trends in global and regional population-weighted annual mean NO_2_ (PWM_NO2_). Approximately 1.6 billion people lived in regions where average NO_2_ significantly decreased (*p* < 0.01). In contrast, 3.2 billion people lived in regions where average NO_2_ significantly increased (*p* < 0.01). The globally averaged PWM_NO2_ increased by 0.9%/year (95% CI: 1.1, 0.6). The four regions with the highest long-term mean PWM_NO2_ are high-income Asia Pacific (Japan, South Korea, Brunei, and Singapore), western Europe, high-income North America (Canada and the United States), and East Asia (China, North Korea, and Taiwan). All have trends significant at *p* < 0.01.

**Table 1:** Satellite-derived population-weighted mean NO_2_ concentrations, linear trends from 1996–2012, and population within Global Burden of Disease Study regions.*^a^*

Region	Mean (1996–2012) NO_2_ (parts per billion)	Trend (percent/year) (95% CI)	*p*‑Value	Human population (millions)
1. Asia Pacific (high-income)	4.9	–2.1 (–2.7, –1.5)	< 0.01	176
2. Western Europe	4.1	–2.5 (–3.0, –2.1)	< 0.01	399
3. North America (high-income)	3.7	–4.7 (–5.3, –4.1)	< 0.01	329
4. East Asia	2.9	6.7 (6.0, 7.3)	< 0.01	1,351
5. Eastern Europe	2.2	1.4 (0.0, 2.9)	0.05	212
6. Central Europe	2.1	–1.4 (–2.1, –0.7)	< 0.01	121
7. Southern Latin America	1.3	–1.0 (–2.4, 0.3)	0.12	59
8. Central Latin America	1.0	–2.0 (–3.0, –1.0)	< 0.01	218
9. Australasia	0.9	–1.5 (–2.2, –0.8)	< 0.01	23
10. Southern sub-Saharan Africa	0.9	–1.2 (–1.8, –0.5)	< 0.01	65
11. North Africa/Middle East	0.7	2.4 (1.8, 3.1)	< 0.01	411
12. Tropical Latin America	0.7	–2.6 (–3.6, –1.6)	< 0.01	189
13. Central Asia	0.6	0.3 (–1.4, 1.9)	0.30	79
14. Andean Latin America	0.6	0.5 (–0.5, 1.4)	0.71	51
15. South Asia	0.5	1.3 (0.4, 2.2)	< 0.01	1,455
16. Southeast Asia	0.5	0.0 (–0.7, 0.7)	0.96	578
17. Caribbean	0.2	–1.1 (–2.2, 0.0)	0.05	25
18. West sub-Saharan Africa	0.2	–0.2 (–1.1, 0.6)	0.56	282
19. Central sub-Saharan Africa	0.1	–1.3 (–2.1, –0.6)	< 0.01	85
20. East sub-Saharan Africa	0.1	–0.1 (–0.9, 0.6)	0.70	301
21. Oceania	< 0.1	–2.9 (–5.4, –0.5)	0.02	7
Global	1.6	0.9 (0.6, 1.1)		6,357
Results are in descending order of mean NO_2_. ^***a***^Lim et al. (2012).				

**Figure 3:**
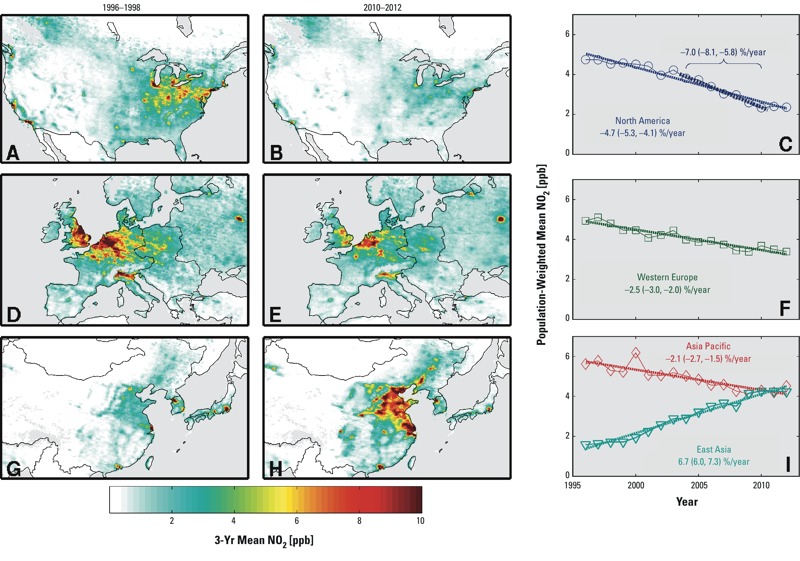
Maps (with regional boundaries) of mean satellite-derived NO_2_ concentrations at the beginning and end of the record for the four regions with the highest long-term population-weighted mean NO_2_: North America (*A*,*B*), western Europe (*D*,*E*), and East China and Asia Pacific (*G*,*H*). Time series of annual population-weighted mean NO_2_ for these regions are shown in the panels at right (*C*,*F*,*I*), with the 1996–2012 linear slopes reported as %/year (relative to the long-term mean), and 95% confidence intervals. The slope from 2004–2010 for North America (relative to the long-term mean) is also shown below the long-term trend.

Figure 3 shows the 17-year annual PWM_NO2_ time series in these four regions; maps of satellite-derived NO_2_ concentrations at the beginning and end of the record are also shown. In Canada and the United States (Figure 3A,B), the satellite-derived estimates of PWM_NO2_ decreased by 50% overall, with a linear trend of –4.7%/year (95% CI: –5.3, –4.1) relative to the long-term mean. The steepest decline occurred between 2004 and 2010, when PWM_NO2_ decreased at a rate of –7.0%/year (95% CI: –5.8, –8.1) relative to the long-term mean. This trend is significantly different from (*p* < 0.01), and almost three times steeper than, the trend of –2.5%/year (95% CI: –4.0, –1.1) for earlier years between 1996 and 2003. The maps of NO_2_ concentrations in North America show that the decreases were most notable over urban areas of the eastern United States and California.

The satellite-derived estimates of PWM_NO2_ in western Europe (Figure 3C,D) also decreased between 1996 and 2012, declining 30% overall, with a linear trend of 2.5%/year (95% CI: –3.0, –2.1). The trend from 1996 to 2003 (–2.9%/year, 95% CI: –5.0, –1.0) was similar to that observed in North America over the same time. However, from 2004 to 2010, the decline in NO_2_ in western Europe (–2.0%/year, 95% CI: –3.9, –0.2) was weaker than that in North America. The maps show notable decreases over the southern United Kingdom, northern France, Germany, and Benelux (comprising Belgium, the Netherlands, and Luxembourg) regions.

The maps in Figure 3E,F encompass both the East Asia and high-income Asia Pacific regions. Before 2010, the highest PWM_NO2_ of all GBD regions was consistently observed for Asia Pacific (South Korea and Japan account for 97% of this population). The region experienced a decrease of 20% from 1996 levels, with a linear trend of –2.1%/year (95% CI: –2.7, –1.5). In contrast, our satellite-derived ground-level estimates indicated that PWM_NO2_ in East Asia increased 2.7 times over the same period of time, at a rate of 6.7%/year (95% CI: 6.0, 7.3), and now has the highest PWM_NO2_ of all GBD regions. China accounts for 97% of this population, and the maps in Figure 3E,F show the notable increase in NO_2_ for eastern China in particular.

The GPW population distribution changed remarkably across East Asia from 1996 to 2012. However, the increase in region-wide PWM_NO2_ was dominated almost entirely by changes in NO_2_ as opposed to changes in population. If the population is held constant at 1996 levels, the trend in PWM is 6.5%/year (95% CI: 5.9, 7.1), well within the 95% CI of the original slope. Similarly, the slopes for western Europe, North America, and Asia Pacific were within the original 95% CIs when population changes were ignored (data not shown).

**Figure 4:**
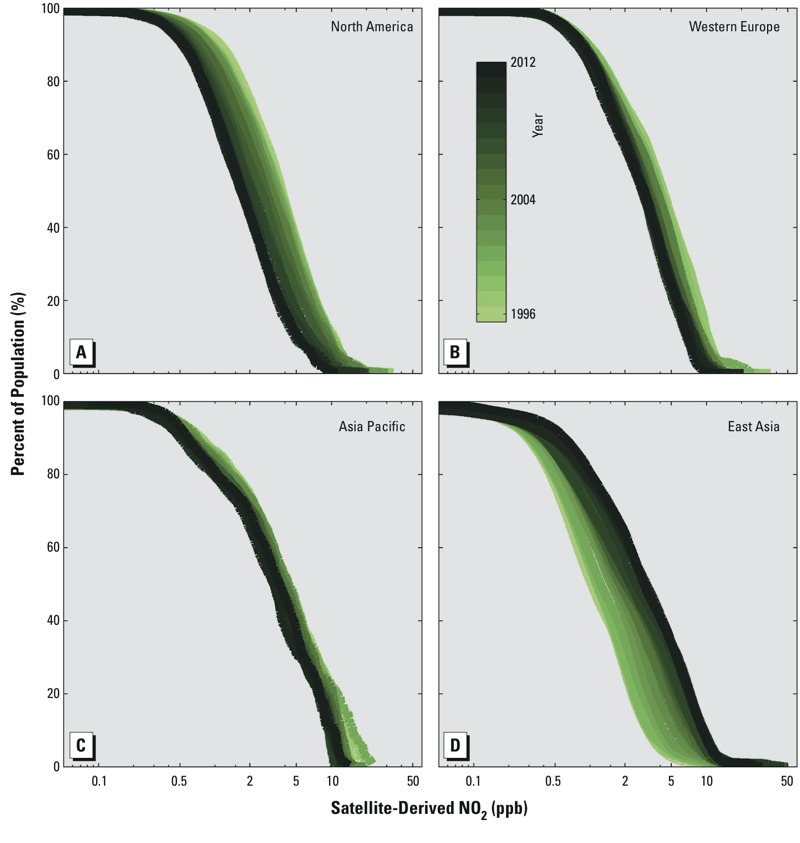
Population and satellite-derived ground-level NO_2_ plotted as cumulative distribution plots for the four regions depicted in Figure 3: (*A*) North America, (*B*) western Europe, (*C*) Asia Pacific, (*D*) East Asia.

*Distribution of population and NO_2_.* Figure 4 presents the population-weighted changes in ambient NO_2_ as cumulative distribution plots for the same four regions depicted in Figure 3. The most extreme ambient concentrations changed locations over time, moving from North America and western Europe in 1996 to East Asia by 2012.

In North America (Figure 4A), the 99th percentile of the population-weighted ambient NO_2_ concentration (highest exposure) decreased by 42% (from 15.7 to 9.0 ppb), and the 50th percentile decreased by 54% (from 3.8 to 1.7 ppb). Concentrations also decreased for less-exposed populations, with the lowest 10th percentile decreasing by approximately 50% (from 1.1 to 0.6 ppb).

In western Europe (Figure 4B), the 99th and 50th percentiles of the population-weighted ambient NO_2_ concentration both decreased by approximately 30% (from 12.9 to 9.1 ppb and 4.1 to 2.9 ppb, respectively). In contrast, the lowest 10th percentile only decreased by approximately 10% (from 1.0 to 0.9 ppb).

In Asia Pacific (Figure 4C), improvements were similarly modest across all segments of the population. The 99th and 50th percentiles of the population-weighted ambient NO_2_ concentration decreased by 16% (from 15.9 to 13.4 ppb) and 12% (4.1 to 3.6 ppb), respectively. The cleanest 10th percentile decreased by 24% (from 0.8 to 0.6 ppb).

In contrast to the other three regions highlighted, NO_2_ concentrations increased for more than 90% of the population in East Asia (Figure 4D). The 99th percentile of the population-weighted ambient NO_2_ concentration more than doubled between 1996 and 2012 (from 8.9 to 21.6 ppb). Similarly, the 50th percentile tripled (from 1.0 to 2.9 ppb). The 10th percentile also doubled (from 0.3 to 0.6 ppb), indicating that conditions deteriorated for both the less-exposed and the highly exposed segments of the population.

*Comparing global ground-level NO_2_ and PM_2.5_ distributions.* Comparing NO_2_ and PM_2.5_ offers insight into the air pollution mixture and into the sources that affect exposure. Table 2 shows satellite-derived estimates of population-weighted PM_2.5_ trends and long-term mean concentrations from van Donkelaar et al. (2014) for the regions investigated in the present study alongside our results for NO_2_ for the same time period (1998–2012).

**Table 2:** Long-term means and trends in satellite-derived population-weighted ground-level mean NO_2_ compared with long-term means and trends in satellite-derived population-weighted PM_2.5_ from van Donkelaar et al. (2014).

Region	Mean NO_2_ (2001–2010) (parts per billion)	1998–2012 trend for NO_2_ (percent/year) (95% CI)	2001–2010 PM_2.5_ (μg/m^3^)(ranking)^*a*^	1998–2012 trend for PM_2.5_ (percent/year) (95% CI)^*a*^
Asia Pacific (high-income)	4.7	–2.1 (–2.9, –1.3)	16.8 (7)	–0.4 (–1.2, 0.4)
Western Europe	3.9	–2.3 (–2.9, –1.8)	13.5 (8)	–1.9 (–2.8, –1.0)
North America (high-income)	3.5	–5.3 (–6.0, –4.6)	9.9 (12)	–3.3 (–4.1, –2.5)
East Asia	3.3	6.5 (5.8, 7.3)	50.3 (1)	3.2 (2.1, 4.3)
Eastern Europe	2.3	1.8 (–0.1, 3.6)	12.6 (9)	–0.3 (–2.0, 1.4)
Central Europe	2.0	–0.8 (–1.6, –0.1)	17.8 (5)	–1.2 (–2.7, 0.3)
Southern Latin America	1.3	–0.6 (–2.3, 1.1)	6.4 (17)	1.3 (–0.1, 2.7)
Central Latin America	1.0	–2.2 (–3.3, –1.1)	8.5 (14)	–0.8 (–1.6, 0)
Australasia	0.9	–1.6 (–2.6, –0.6)	3.0 (20)	0.3 (–0.7, 1.3)
Southern sub-Saharan Africa	0.9	–1.6 (–2.3, –0.9)	5.9 (18)	1.5 (0.1, 2.9)
North Africa/Middle East	0.8	2.5 (1.7, 3.2)	25.5 (4)	1.5 (0.7, 2.3)
Tropical Latin America	0.6	–3.0 (–4.3, –1.7)	5.0 (19)	0.2 (–0.6, 1.0)
Central Asia	0.6	0.5 (–0.6, 1.7)	17.3 (6)	1.7 (0.7, 2.7)
Andean Latin America	0.6	–0.0 (–2.1, 2.0)	6.6 (16)	1.4 (–0.7, 3.5)
South Asia	0.5	1.7 (0.7, 2.7)	34.6 (2)	2.9 (2.2, 3.6)
Southeast Asia	0.5	–0.1 (–1.0, 0.8)	11.0 (11)	2.7 (1.9, 3.5)
Caribbean	0.2	–0.8 (–2.2, 0.6)	7.0 (15)	–0.3 (–1.3, 0.7)
West sub-Saharan Africa	0.2	–0.4 (–1.5, 0.7)	30.8 (3)	–0.1 (–1.0, 0.8)
Central sub-Saharan Africa	0.1	–1.7 (–2.7, –0.8)	11.4 (10)	–0.4 (–1.2, 0.4)
East sub-Saharan Africa	0.1	–0.3 (–2.1, 1.4)	9.8 (13)	1.0 (0.1, 1.9)
Oceania	< 0.1	–0.4 (–2.1, 1.4)	2.3 (21)	3.9 (2.6, 5.2)
Global	1.7	1.0 (0.74, 1.3)	26.4	2.1 (1.6, 2.6)
Numbers in parentheses for mean PM_2.5_ give regional ranking. ^***a***^From van Donkelaar et al. (2014).				

East Asia had the highest long-term population-weighted concentrations of PM_2.5_ (50.3 μg/m^3^, Table 2) but ranked fourth for NO_2_ (3.3 ppb). In contrast, whereas Asia Pacific ranked highest in terms of NO_2_ (4.7 ppb), it ranked seventh for PM_2.5_ (16.8 μg/m^3^). South Asia experienced the second-highest ambient concentrations of PM_2.5_ (34.6 μg/m^3^), yet this region only ranked fifteenth for NO_2_ (0.5 ppb). Another significant shift in ranking occurred for North America, whose population was exposed to the world’s third-highest ambient concentrations of NO_2_ (3.5 ppb) but ranked much lower (twelfth) in terms of PM_2.5_ (9.9 μg/m^3^).

Table 2 shows that of all the GBD regions, North America had the steepest declines in ambient concentrations of both NO_2_ (–5.3%/year, 95% CI: –6.0, –4.6) and PM_2.5_ (–3.3%/year, 95% CI: –4.1, –2.5). The only other region with statistically significant declines in both was western Europe. Of the remaining seven regions with significant decreasing trends for NO_2_, none had significant changes in PM_2.5_: most notably, Asia Pacific. In contrast, PM_2.5_ increased significantly in eight regions, but only three of these had significant increases in NO_2_ as well (East Asia, North Africa/Middle East, and South Asia). Southern sub-Saharan Africa is the only region where statistically significant trends were in opposing directions (decreasing NO_2_, increasing PM_2.5_).

*Uncertainty in the satellite-derived trends.* We used the comparison with ground-station observations across North America and Europe to assess the error in our regional satellite-derived trends. The confidence intervals of the satellite-derived trends (± 0.6%/year over North America and Europe from the last two panels of Figure 2) roughly describe the difference between the satellite-derived and *in situ* trends (0.7%/year for North America, 0.3%/year for Europe). Thus, we treated the confidence intervals as a proxy measure of error in the relative trends for which there were no ground-based observations. The steeper slope derived from the comparison of the regional averaged satellite observations with the ground-based measurements (last two panels of Figure 2) implies that there could be a small systematic bias in the satellite-derived trends, but this may be attributable to representativeness differences in the observed quantities (spatial average from the satellite vs. point measurements by the ground monitors).

Sampling losses in the satellite record caused by quality control filtering contributed to systematic error in the annual mean. For 13 of the 21 regions, our correction for incomplete sampling changed the calculated PWM_NO2_ by < 25%. However, in the northern latitudes with seasonal snow cover, the correction term became large owing to the seasonality of NO_2_ concentrations. The correction was largest for western Europe and increased long-term PWM_NO2_ by 60% (from 2.6 to 4.1 ppb). Globally, the correction increased PWM_NO2_ by 33% (from 1.2 to 1.6 ppb).

We tested the sampling correction by comparing the linear regression slopes with and without adjusting for sampling bias. In all cases, the sampling correction had a statistically nonsignificant impact on the regional trends in PWM_NO2_. For example, the trend over North America without the sampling correction was –4.7%/year (95% CI: –5.5, –3.9) versus –4.7%/year (95% CI: –5.3, –4.1) with the correction. Similarly, in western Europe, where the correction was largest, the trend without the correction was –2.4%/year (95% CI: –3.3, –1.4) versus –2.5%/year (95% CI: –3.0, –2.1) with the correction. Globally, the trend without the sampling correction was 1.3%/year (95% CI: 0.9, 1.6) versus 0.9%/year (95% CI: 0.6, 1.1) with the correction (*p* = 0.04).

Although other systematic errors in annual mean NO_2_ are expected to have less influence on the trend calculation, and estimating trends over large regions reduces random error across space, we caution that errors in annual NO_2_ over any individual 0.1° × 0.1° pixel will be large. Error in estimating ground-level NO_2_ from satellite observations (because of errors in the simulated profile and in the retrieved column densities) has been previously estimated as –11% to +36% (Lamsal et al. 2008). Error introduced by simulating spatial resolution in the GOME and GOME-2 observations could also be large over an individual pixel, although we found no perceptible bias resulting from our approach in the comparison with ground-based observations for large samples. Given this evidence, we expect the error in estimating ground-level NO_2_ is random and can be mitigated by spatial averaging.

## Discussion

NO_2_ acts as an indicator for exposure to unmeasured toxins in the air pollution mix (Brook et al. 2007) and continues to have strong associations with negative health outcomes (MacIntyre et al. 2014). However, traditional ground-station NO_2_ monitoring networks have poor spatial coverage, leading to large gaps in assessments of human exposure (Guay et al. 2011), particularly in developing countries. We found that satellite observations can be used to derive global long-term changes in ambient NO_2_ concentrations at spatial scales relevant to average human exposure (0.1° × 0.1°). The record offers almost complete spatial coverage, not only supplementing gaps in established ground-station observing networks but also providing insight into regions without prior observations.

We used our satellite-derived ground-level NO_2_ concentrations to calculate worldwide trends in population-weighted annual mean NO_2_ from 1996 to 2012. Decreasing trends were found in North America (–4.7%/year), western Europe (–2.5%/year), and Asia Pacific (–2.1%/year). Increasing trends were found in East Asia (6.7%/year), North Africa/Middle East (2.4%/year), and South Asia (1.3%/year). The trend in global PWM_NO2_ was increasing (0.9%/year) and statistically significant. Our calculated trends in PWM_NO2_ were consistent with ground-based measurements in North America and Europe and with previous work describing changes in NO_2_ tropospheric columns over North America (e.g., Duncan et al. 2013; Russell et al. 2012) and China (Mijling et al. 2013; Richter et al. 2005), and over the megacity regions studied by Hilboll et al. (2013). Our satellite-derived time series is freely available (http://fizz.phys.dal.ca/~atmos/martin/?page_id=232).

The regional trends reflected differing trajectories of anthropogenic activities and policies around the world. For example, increases in PWM_NO2_ for East Asia and for the Middle East from 1996 to 2012 reflect rapid development in regions where emissions have been rising but remain uncertain (e.g., Zhang et al. 2007). In contrast, the modest decline in PWM_NO2_ in North America between 1996 and 2003, which was followed by steeper decreases post-2004, might have resulted from the U.S. EPA’s “NO_x_ State Implementation Plans.” These plans required NO_x_ (nitrogen oxides) emission reduction measures to be implemented by mid-2003 and for total anthropogenic emissions in the United States to decrease by 26% between 2004 and 2009 (U.S. EPA 2014). The steeper decline in PWM_NO2_ that ocurred during this period (40%) compared with the emissions reported by the U.S. EPA and with the decrease in area-weighted mean NO_2_ (25%, not shown), suggests that emission reductions successfully and most efficiently targeted air quality in populated regions. Other factors, including the 2008 economic recession (Castellanos and Boersma 2012; Russell et al. 2012), may have also played a role in the reduction.

The regional rankings of population-weighted NO_2_ concentrations, and the long-term trends, differed from previously reported values for PM_2.5_ (van Donkelaar et al. 2014), suggesting that these observations provide complementary information on the changing global air pollution mixture. For example, some regions that ranked highest in terms of PM_2.5_ (e.g., East Asia and South Asia) ranked low for NO_2_. A driver of these differences lies in the types of combustion that produce NO_2_ and PM_2.5_, with biofuel sources in South Asia and intense coal burning in East Asia (Lu et al. 2011). We also found regions where NO_2_ significantly declined but PM_2.5_ was not significantly different (e.g., Asia Pacific). Comparing epidemiologic studies of the various regions could yield insight into the health effects of different pollution source types.

Caution must be taken when comparing the satellite-derived ground-level NO_2_ concentrations we obtained with the WHO air quality guidelines for NO_2_ because NO_2_ exhibits dramatic within-city spatial variation (Jerrett et al. 2005; Wang et al. 2014), which these satellite-derived data cannot fully resolve. For example, this data set is not appropriate for determining gradients in exposure near roadways. Plumes from stack emissions could result in additional errors. Our estimates represent area averages (~ 10 km × 10 km), whereas the WHO guideline was established for point measurements that are often not representative of area averages. We hypothesize that the area-averaged record from satellites represents what a randomly distributed monitoring network would obtain, potentially mitigating systematic biases in studies of exposure that result from station placement and poor network coverage (Goldman et al. 2010; Sheppard et al. 2012). We have focused on long (annual) averaging times to minimize the daily variability in the relationship between column-to-surface concentrations (Knepp et al. 2013) that may not be fully captured by the chemical transport model. These data are therefore also better suited to long-term than to short-term studies.

## Conclusion

We reported trends in population-weighted averages to assess changes in NO_2_ around the world, selecting regions according to the Global Burden of Disease Study for a convenient grouping of nations based on “geographic closeness and epidemiological similarities” (http://www.healthdata.org/gbd/faq). These regional population-weighted averages are indirectly related to average human exposure. Personal exposure would need to account for additional factors such as individual activity patterns and indoor versus outdoor contributions.

Multiple opportunities are anticipated for further development. We expect improvements in this approach from the increasing resolution of future satellite NO_2_ observations and from higher-resolution modeling of the relationship between ground-level concentrations and the observed column densities. Land use regression could offer additional valuable fine-scale information (Novotny et al. 2011; Vienneau et al. 2013). Forthcoming geostationary platforms dedicated to monitoring air quality [e.g., TEMPO (http://science.nasa.gov/missions/tempo/), Sentinel-4 (https://sentinel.esa.int/web/sentinel/missions/sentinel-4), and GEMS (Lasnik et al. 2014)] will also significantly increase temporal coverage. Our results add complementary information to the assessment of population exposure to air pollution.

## Supplemental Material

(1.3 MB) PDF
