# Supplementary material for: Long-Term Trends Worldwide in Ambient NO2 Concentrations Inferred from Satellite Observations
Source: Environ Health Perspect. 2015 Aug 4;124(3):281–9. doi: 10.1289/ehp.1409567 (PMC4786989; doi:10.1289/ehp.1409567)
Supplement: (1.3 MB) PDF [file ehp.1409567.s001.acco.pdf]

Environ Health Perspect
DOI: 10.1289/ehp.1409567

Note to Readers: *EHP* strives to ensure that all journal content is accessible to all readers. However, some figures and Supplemental Material published in *EHP* articles may not conform to 508 standards due to the complexity of the information being presented. If you need assistance accessing journal content, please contact ehp508@niehs.nih.gov. Our staff will work with you to assess and meet your accessibility needs within 3 working days.

Supplemental Material

Long-Term Trends Worldwide in Ambient NO_2 Concentrations Inferred from Satellite Observations

Jeffrey A. Geddes, Randall V. Martin, Brian L. Boys, and Aaron van Donkelaar

Table of Contents

Figure S1: Evaluation over North America of the global approach to combine multiple satellite instruments. (a) Unaltered GOME-derived ground-level NO_2 observations from 1999 and (b) downscaled GOME observation based on relative spatial structure observed with SCIAMACHY from 2003-2005. (c) Mean SCIAMACHY-derived ground-level NO_2 from 2003-2005, and (d) from 2009-2011. (e) SCIAMACHY observations from 2009-2011 smoothed to represent GOME horizontal resolution, and (f) downscaled SCIAMACHY observations for the same time period using the SCIAMACHY-derived ratio of high resolution to coarse resolution during 2003-2005. Agreement between (d) and (f) is high (r = 0.96).

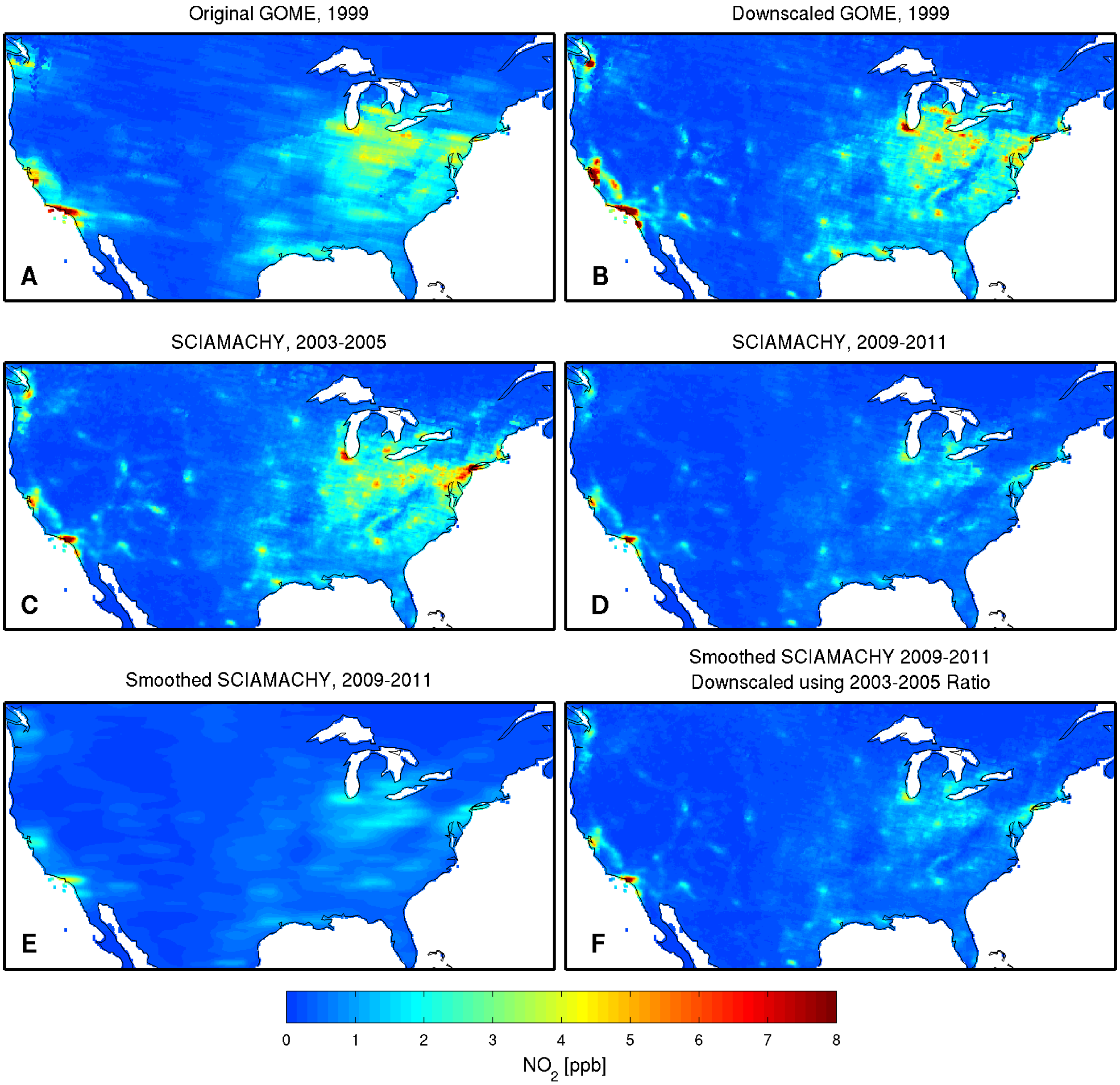

Figure S1: Evaluation over North America of the global approach to combine multiple satellite instruments. (a) Unaltered GOME-derived ground-level NO_2 observations from 1999 and (b) downscaled GOME observation based on relative spatial structure observed with SCIAMACHY from 2003-2005. (c) Mean SCIAMACHY-derived ground-level NO_2 from 2003-2005, and (d) from 2009-2011. (e) SCIAMACHY observations from 2009-2011 smoothed to represent GOME horizontal resolution, and (f) downscaled SCIAMACHY observations for the same time period using the SCIAMACHY-derived ratio of high resolution to coarse resolution during 2003-2005. Agreement between (d) and (f) is high (r = 0.96).
